# Opticin, a small leucine-rich proteoglycan, is uniquely expressed and translocated to the nucleus of chronic lymphocytic leukemia cells

**DOI:** 10.1186/2162-3619-2-23

**Published:** 2013-08-28

**Authors:** Eva Mikaelsson, Anders Österborg, Zahra Tahmasebi Fard, Ahmad Mahmoudi, Jafar Mahmoudian, Mahmood Jeddi-Tehrani, Mehdi Akhondi, Fazel Shokri, Paul N Bishop, Hodjattallah Rabbani, Håkan Mellstedt

**Affiliations:** 1 Immune and Gene Therapy Lab, CCK, Department of Oncology-Pathology, Karolinska Institute, Stockholm, Sweden; 2 Departments of Oncology (Radiumhemmet) and Hematology, Karolinska University Hospital Solna, Stockholm, Sweden; 3 Monoclonal Antibody Research Center, Avicenna Research Institute, ACECR, Tehran, Iran; 4 Reproductive Biotechnology Research Center, Avicenna Research Institute, ACECR, Tehran, Iran; 5 Department of Immunology, School of Public Health, Tehran University of Medical Sciences, Tehran, Iran; 6 Centre for Hearing and Vision Research, Institute of Human Development, University of Manchester and Central Manchester University Hospitals NHS Foundation Trust, Manchester Academic Health Science Centre, Manchester, UK; 7 Department of Biology, Roudehen Branch, Islamic Azad University, Roudehen, Iran

**Keywords:** Chronic lymphocytic leukemia, Opticin, Nucleus, Core protein, Proteoglycans

## Abstract

**Background:**

Opticin (OPTC) is a member of the small leucine-rich proteoglycan (SLRP) family and is localized particularly in certain extracellular matrices. We have previously reported the unique expression of another SLRP, fibromodulin (FMOD) in the leukemic cells of patients with chronic lymphocytic leukemia (CLL). *OPTC* is located in the same region as *FMOD* on chromosome 1 (1q32.1). Cluster up-regulation of genes may be observed in malignancies and the aim of the present study was to analyze the expression of OPTC in CLL cells.

**Methods:**

The expression of OPTC was tested by RT-PCR and realtime qPCR in PBMC from CLL patients, other hematological malignancies and healthy controls. The presence of OPTC protein, and its subcellular localization, was investigated using fractionation methods where the obtained lysate fractions were analyzed by Western blotting. Deglycosylation experiments were performed to investigate the glycosylation status of the CLL OPTC.

**Results:**

*OPTC* was expressed at the gene level in all patients with CLL (n = 90) and in 2/8 patients with mantle cell lymphoma (MCL) but not in blood mononuclear cells of healthy control donors (n = 20) or in tumor samples from nine other types of hematological malignancies. OPTC was detected by Western blot in all CLL samples analyzed (n = 30) but not in normal leukocytes (n = 10). Further analysis revealed a CLL-unique unglycosylated 37 kDa core protein that was found to be located preferentially in the cell nucleus and endoplasmic reticulum (ER) of the CLL cells.

**Conclusions:**

A 37 kDa unglycosylated OPTC protein was detected in ER and in the nucleus of CLL cells and not in healthy control donors. The function of this OPTC core protein remains unclear but its CLL-specific expression and subcellular localization warrants further investigations in the pathobiology of CLL.

## Introduction

Chronic lymphocytic leukemia (CLL) is the most common adult leukemia and has a highly variable clinical course. It is characterized by an accumulation of B cells expressing the cell surface antigens CD5, CD19, CD23, CD20_dim_ and sIgM_dim_. In the last years, there has been an increased interest to characterize cellular and molecular mechanisms involved in the CLL biology with the aim to develop new targeted therapeutics [1,2].

In a study by Klein et al., the global gene expression profiling of CLL cells showed an increased expression of the extracellular matrix (ECM) protein fibromodulin (FMOD) [3]. FMOD is a member of the small leucine-rich proteoglycan (SLRP) family and is normally expressed in collagen-rich tissues. We demonstrated that FMOD was expressed at the gene and protein levels in CLL and mantle cell lymphoma (MCL) cells [4]. The unexpected finding of an ECM protein in leukemic cells raised the question whether also other SLRP family members might be expressed in CLL cells.

Cluster/locus upregulation of genes has been reported in malignant diseases [5]. FMOD is located on chromosome 1q32.1 [6], adjacent to two other members of the SLRP family: proline/arginine-rich end leucine-rich repeat protein (PRELP) and opticin (OPTC). OPTC was first described as a 90 kDa homodimeric glycoprotein in bovine vitreous [7,8] but in addition, opticin glycoprotein has been detected in cartilage [9] and OPTC mRNA expression has been noted in cartilage, brain, ligament, testis, muscle and skin [7,10-12]. OPTC interacts with collagen, growth hormone and heparin sulphate and may play a role in modulating cell anchorage, growth factor sequestration as well as regulation of angiogenesis [13-16].

In this study we analyzed the expression of OPTC in CLL cells as an extension to our previous studies on FMOD [4] in CLL. We found that OPTC was expressed as an unglycosylated core protein in the nucleus and ER of CLL cells and not in healthy control donors or other hematological malignanices.

## Results

### OPTC gene expression

**Figure 1:**
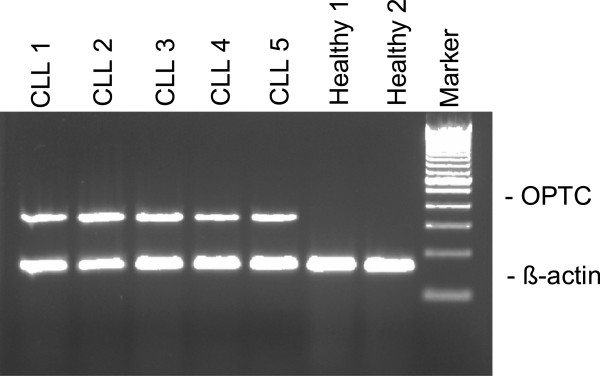
**OPTC gene expression (RT-PCR) in PBMC of CLL (n = 5) and healthy controls donors (n = 2).** β-actin was used to verify the integrity of synthesized cDNA. Marker is a 100 bp DNA ladder.

The expression of OPTC mRNA in tumor cells from CLL patients and nine other types of hematological malignancies as well as PBMC and leukocyte subsets from healthy controls donors was tested by RT-PCR. PBMC of all CLL patients (n = 90) expressed OPTC at the mRNA level irrespective of clinical phase (non-progressive / progressive). OPTC was also expressed in tumor cells of some MCL patients (2/8) but not in tumor cells from other hematological malignancies e.g. AML, CML, PLL, HCL, FL, lymphoplasmacytoid lymphoma, MM, and ALL. Neither was OPTC detected in normal PBMC (n = 20) or isolated B cells (purity >90%, n = 3) and T cells (purity > 70%, n = 3) of healthy control donors (Table 1; Figure 1). OPTC was not expressed in any of the six CLL cell lines tested (EHEB, I83,Wa, CII, 232B4, PGA) (data not shown).

**Table 1:** **OPTC gene expression** (**RT**-**PCR**) **in isolated tumor cells from patients with various types of hematological malignancies and normal leukocyte subsets of healthy control donors**

**Cell source**	**No. of positive cases/total no.**
Chronic lymphocytic leukemia (PBMC)	90/90
Mantle cell lymphoma (PBMC)	2/8
Pre B-ALL (PBMC)	0/1
Chronic myelogenous leukemia (PBMC)	0/7
Acute myelogenous leukemia (PBMC)	0/7
Prolymphocytic leukemia (B and T cell types) (PBMC)	0/3
Hairy cell leukemia (PBMC)	0/2
Lymphoplasmacytoid lymphoma (PBMC)	0/2
Multiple myeloma (BMMC)	0/6
Follicular lymphoma (PBMC*)	0/2
Normal healthy PBMC (lymphocytes and monocytes)	0/20
Normal blood T cells**	0/3
Normal blood B cells***	0/3

*PBMC* peripheral blood mononuclear cells, *BMMC* bone marrow mononuclear cells.

* Severe lymphocytosis.

** Purity >70%, *** purity >90%.

**Figure 2:**
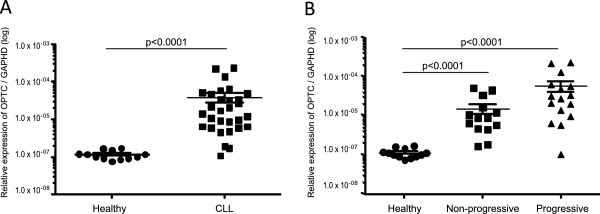
**Quantitative OPTC gene expression (q-PCR) in PBMC of CLL patients (non-progressive/progressive) and healthy controls. A** The relative expression of OPTC was significantly higher in CLL patients (n = 30) compared to healthy control donors (n = 12) (p < 0,0001). **B** The relative expression of OPTC was higher in progressive (n = 16) and non-progressive (n = 14) patients compared to healthy donors (p < 0,0001). There was no significant difference in OPTC expression between non-progressive and progressive CLL. Relative expression of OPTC was calculated using the ΔCt method and GAPDH as reference gene.

The relative expression of OPTC was quantified in relation to the housekeeping gene GAPDH using qPCR. The expression of OPTC was significantly higher in CLL patients (n = 30) compared to healthy control donors (n = 12) (p < 0,0001). Comparing CLL samples from progressive and non-progressive patients showed a higher relative expression of OPTC in each of the two groups compared to healthy donors (p < 0,0001 respectively). The mean relative expression of OPTC appeared to be somewhat higher in progressive (n = 16) than non-progressive (n = 14) CLL patients but the difference was statistically not significant (Figures 2A and 2B).

### Specificity of anti-OPTC antibodies

**Figure 3:**
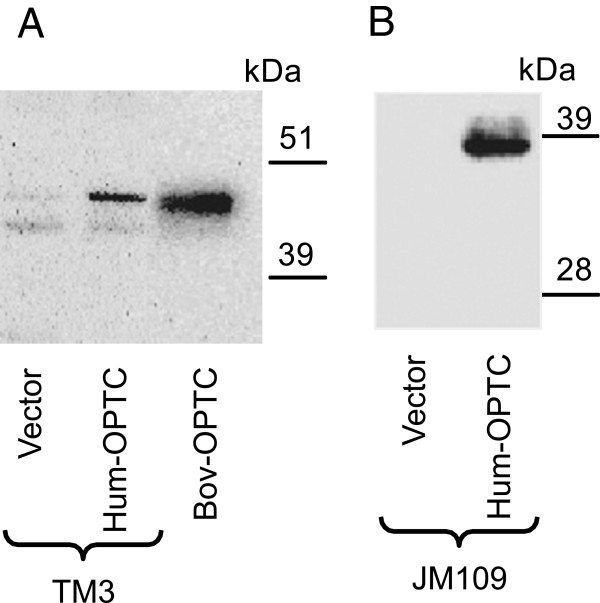
**Western blot analyses to validate the specificity of the own-produced anti-OPTC antibody.** The precursor OPTC gene was cloned into pcDNA3.1+ vector and transfected into **A)** mouse TM3 cell line and **B)** E. coli JM109. **Lane 1**: Lysate of cells transfected with the vector alone (negative control). **Lane 2**: Lysate of cells transfected with the OPTC construct. **Lane 3**: bovine OPTC produced in HEK-293 cells. Our C-terminal pAb detected a 48 kDa human OPTC expressed in TM3 cells, a 37–38 kDa OPTC produced in bacteria JM109 and a 45–50 kDa recombinant bovine OPTC.

The molecular weight by SDS-PAGE of the normal glycosylated OPTC protein varies between 45–50 kDa [7,11]. The specificity of our C-terminal anti-OPTC pAb was tested in Western blot against recombinant bovine OPTC and human OPTC expressed in *E*.*coli* and TM3 mouse cells. Cells transfected with the vector alone were used as a negative control. Bovine OPTC was recognized as a 45–50 kDa band (Figure 3A), corresponding to the mature, glycosylated OPTC protein [11]. Human OPTC expressed in the TM3 mouse cell line, had a molecular mass of 48 kDa (Figure 3A) while OPTC expressed in bacteria JM109 was noted as a 37–38 kDa band (Figure 3B).

### OPTC protein expression in CLL cells

**Figure 4:**
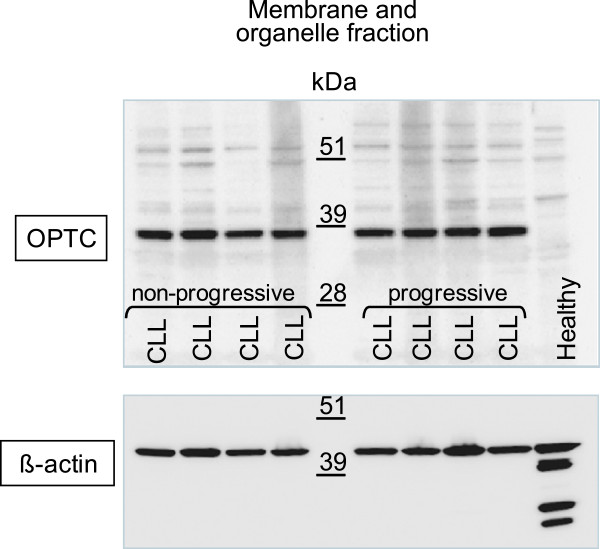
**Western blot of cell lysates from CLL patients and healhty control donors.** The membrane/organelle fraction from non-progressive (n = 4) and progressive (n = 4) CLL patients and from one healthy control. *Upper panel*: a rabbit anti- C-terminal OPTC pAb detecting a 37 kDa band in the membrane/organelle fraction of CLL cells but not in healthy donor PBMC. *Lower panel*: The same membrane stripped and re-probed with an anti-β-actin mAb. Multiple bands were detected in the healthy control while in CLL patients a single 42 kDa band was noted.

PBMC of CLL patients (n = 30) were tested for OPTC protein expression by Western blot. Leukemic cell lysates were prepared by a 2-step method giving rise to two fractions. In the lower, Triton X-resistant fraction containing membrane and organelle proteins [17], a 37 kDa band was seen in all CLL patients (n = 30) irrespective of clinical phase (non-progressive / progressive) but not in any of the healthy controls (n = 10) (Figure 4).

The 37 kDa band was recognized both by the C-terminal and the N-terminal pAbs indicating that the 37 kDa protein was probably not a degradation or cleavage product. No OPTC band could be detected in the upper fraction, holding cytosolic proteins (data not shown).

To ensure equal loading of samples, the membranes were stripped and re-probed with an anti-β-actin mAb (Sigma). In the upper fraction, a band of 42 kDa was detected in CLL patients as well as in healthy controls (data not shown). In the Triton-X resistant fraction, multiple β-actin bands (possible actin turnover cleavage products [18] were noted in the healthy controls whereas CLL samples showed a single 42 kDa band (Figure 4, lower panel).

### Subcellular fractionation

**Figure 5:**
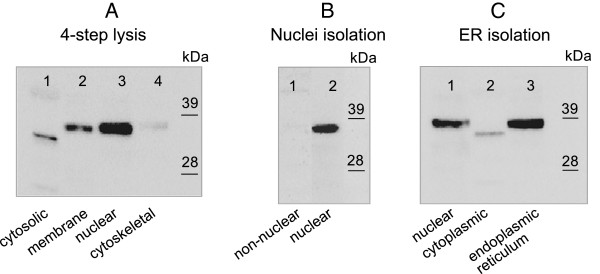
**Western blot of the cell fractionation experiments A) 4-step lysis, B) nuclei isolation and C) ER isolation.** Using the C-terminal, anti-OPTC pAb, a 37 kDa band was detected in membrane, nuclear, and ER fractions (lanes A2, A3, B2, C1 and C3). A band of slightly smaller molecular weight (possible degradation/cleavage fragment) was detected in the cytosol fractions (lanes A1 and C2).

To further analyze the cellular localization of OPTC in CLL cells, we used a four-step lysis procedure in which the CLL cell proteins were fractionated into cytosolic, membrane, nuclear and cytoskeletal proteins respectively. The CLL-specific 37 kDa OPTC band was detected in the membrane as well as in the nuclear fractions. In addition, a band of slightly smaller molecular weight was detected in the cytosolic fraction (Figure 5A). This band was not detected by the N-terminal pAb and is possibly a cleavage product [8].

### Nuclei isolation / localization

A nuclei protein isolation kit (separating nuclear proteins from non-nuclear proteins) was used to analyze nuclear localization of CLL OPTC. The 37 kDa CLL OPTC was detected in the nuclear fraction while the non-nuclear fraction was negative (Figure 5B).

### Endoplasmic reticulum isolation / localization

An endoplasmic reticulum isolation kit was used to isolate ER proteins from CLL cells. The 37 kDa CLL related OPTC was detected in the 1000 g and 100 000 g centrifugation pellets, representing the nuclei and ER fraction respectively. As in the 4-step lysis described above, a weak band of smaller molecular weight (possibly a cleavage product) was noted in the supernatant (12000 g + 100000 g) representing cytoplasmic proteins (Figure 5C).

### Deglycosylation of the OPTC protein

**Figure 6:**
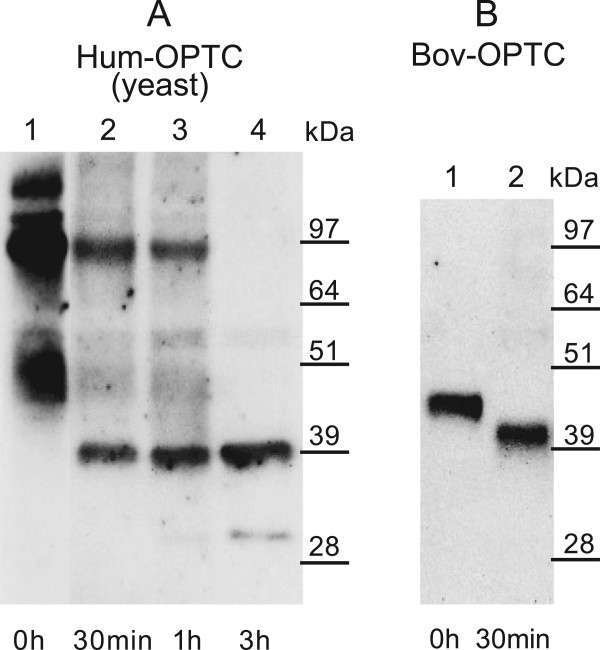
**Western blot of human and bovine OPTC after chemical deglycosylation by TMFS. A)** recombinant human OPTC produced in yeast and **B)** recombinant bovine OPTC. Western blot was performed using a C-terminal anti-OPTC pAb. **Lane 1**: Untreated recombinant OPTC. **Lane 2**: Recombinant OPTC treated with TFMS for 30 min. **Lanes 3 and 4**: Recombinant OPTC treated with TFMS and anisole for 1 and 3 h, respectively. Complete removal of the carbohydrate structures yielded a 39 kDa and a 40–42 kDa band in the human and bovine OPTC, respectively.

To explore structural properties of the 37 kDa OPTC in CLL, chemical deglycosylation was performed using OPTC expressed in yeast and a recombinant bovine OPTC produced in 293-EBNA cells. Yeast-derived OPTC was seen as smeary bands at 45–50 kDa and 70–120 kDa. After 30 min of TFMS treatment, a 39 kDa band appeared while the 45–50 kDa bands had disappeared. The 70–120 kDa bands were reduced in intensity and to a size of 100 kDa. The deglycosylation procedure was enhanced by the use of TFMS and anisole for 1 and 3 hours. After 3 h, the 100 kDa band completely disappeared and the intensity of the 39 kDa band increased. The 39 kDa band may represent completely deglycosylated OPTC (Figure 6A). Also after 3 h, a weak band of 30 kDa appeared, which is believed to be a degradation/cleavage product [8].

Treatment of recombinant bovine OPTC using TFMS for 30 min resulted in the reduction of the protein size from 45–48 kDa to 40–42 kDa (Figure 6B).

## Discussion

In the present study we demonstrated that OPTC, a member of the SLRP family, was overexpressed in leukemic cells from CLL and MCL patients. Tumor cells of other hematological malignancies and PBMC of healthy donors did not express OPTC when tested by RT- and qPCR. Furthermore, an unglycosylated 37 kDa OPTC protein was found to be expressed in CLL cells but not in normal leukocytes.

Normal OPTC is a glycosylated protein that runs at 45–50 kDa on SDS-PAGE gels and is secreted into the ECM compartment of various tissues. The expression has mostly been associated with the eye but has also been identified in other tissues including brain, skin, ligament and cartilage [7,10-12]. In the vitreous humour of the eye extracellular OPTC protein has been shown to be anti-angiogenic by binding to collagen and inhibiting α2β1 and α1β1 integrin-mediated endothelial cell adhesion to the collagen; there is also evidence that it binds growth hormone and may modulate its function [14-16].

There are several reports on SLRPs being overexpressed in cancer [19-22]. They may be expressed either by the tumor cells or by non-cancerous fibroblasts in the surrounding matrix. Several in vitro and in vivo studies suggest that SLRPs may act as antagonists of tumor growth, metastatic spreading and angiogenesis [23].

In this study, OPTC was expressed in the leukemic cells of all CLL patients and in the lymphoma cells of some patients with MCL. There are few reports connecting OPTC expression to cancer. In ciliary body tumor cells (originating from the eye), OPTC was shown to be downregulated compared to normal ciliary body epithelium [24]. In a panel of breast cancer cell lines, OPTC expression was induced by adenoviral transfection, which suppressed proliferation, increased apoptosis and reduced migration [25].

We have previously reported a similar expression pattern for FMOD, a closely related member of the SLRP family [4]. FMOD, OPTC and the other SLRPs are secreted proteins that bind to cell membrane receptors or ECM proteins. However, there are reports of SLRPs with intracellular localization, both in normal and in tumor tissues. FMOD was detected in the cytoplasm of human epidermal keratinocytes [26]. Lumican, another member of the SLRP family, was localized in the cytosol of lung as well as colorectal cancer cells [27].

Using the three cell fractionation kits, OPTC was detected in the nuclear and ER fractions resp. of the CLL cells. The Nuclei isolation kit detected the 37 kDa OPTC in the nuclear fraction. No OPTC protein was noted in the non-nuclear fraction (where the ER OPTC might have been seen) which may be due to large volume of the non-nuclear fraction diluting the OPTC protein. As the focus was on the nuclear OPTC, we did not concentrate the non-nuclear fraction.

Nuclear localization has been observed for some SLRPs (e.g. PRELP, biglycan and decorin) where they might participate in various nuclear processes [28-30]. Decorin was noted in the cytoplasm as well as in the nuclei of human oral cancer biopsies and cell lines [30]. Several decorin variants were found, one of them had a molecular weight corresponding to a decorin protein core without post-translational modifications. The identified nuclear decorin was suggested to play a role in migration and invasion of the malignant cells [31].

Glycosylation is an important process affecting folding, stability and functions of a protein. In normal tissues, SLRPs show varying degrees of glycosylation, which may differ between species and are of importance for tissue distribution and functions [32].

In tumors, the glycosylation of SLRPs may vary between and within individual tumors [21,33-35]. The difference between normal human OPTC (45–50 kDa) and CLL-derived OPTC (37 kDa) may be due to differences in glycosylation. Complete deglycosylation of yeast-derived human OPTC yielded a 39 kDa OPTC, slightly larger than the OPTC detected in CLL cells and the recombinant human OPTC derived of bacteria (37 kDa). The small difference in the molecular weight might be due to other modifications of the yeast derived protein. The 37 kDa protein in CLL may represent the core OPTC protein without side-chain glycan modifications. A 37 kDa OPTC protein with no post-translational modifications has been found in normal rat [11].

The 37 kDa CLL-OPTC was detected both by a C- and a N-terminal anti-OPTC antibody, indicating that this protein may not be a degradation or cleavage product. Thus, CLL cells seemed to express an unmodified protein backbone.

Regardless of glycosylation, the core protein of several SLRPs may express binding sites for interactions with other proteins, e.g. collagen, fibronectin, TGF-β, TNF-α, epidermal growth factor receptor (EGFR) and integrin receptors [36-41]. When coated on Transwell® chambers, recombinant lumican core protein was shown to bind and inhibit cell migration of α2β1 integrin bearing cells [42]. Systemic delivery of decorin protein core, downregulated the epidermal growth factor receptor (EGFR), leading to growth inhibition as well as increased apoptosis of squamous cell carcinoma xenografts in mice [41]. Whether the 37 kDa core OPTC protein in CLL has similar capabilities needs to be further studied.

The biological function of OPTC in CLL is not clear but might resemble those of other SLRPs in malignant cells, such as lumican [42], or decorin [31]. The specific and unique expression of the 37 kDa core OPTC protein in all patients may indicate a functional role. The specific expression of two other proteoglycans, FMOD [4] and PRELP (to be published) in CLL might support a role of proteoglycans in CLL. Functional characterization of the CLL-associated OPTC variant may assist in exploring the role of OPTC in the pathobiology of CLL.

## Conclusions

We demonstrate in this study the unexpected expression of the ECM protein OPTC in all CLL patients and some patients with MCL. The detected OPTC protein was localized to the nucleus and ER of the leukemic cells and had a molecular weight corresponding to an unglycosylated core protein. The unique expression of an unglycosylated OPTC with aberrant localization in CLL cells may suggest involvement in the pathobiology of CLL.

## Materials and methods

### Patients and controls

The diagnosis and clinical staging of CLL (progressive/non-progressive) and other hematological malignancies was established using the WHO classification of hematopoetic and lymphoid malignancies and the IWCLL criteria [43,44]. The CLL patients included 63 men and 27 women who were 44–87 years old at the time of diagnosis. Most patients (77%) were in the age range of 60–79 years. The distribution of Rai stages was as follows: 35 patients (39%) stage 0, 23 patients (26%) stage I, 6 patients (7%) stage II, 23 patients (26%) stage III and 3 patients (3%) stage IV. Progressive disease was observed in 42 patients (47%), and non-progressive disease in 48 patients (53%). 60 patients (67%) were untreated while 30 patients (33%) were previously treated (Table 2). The healthy control group consisted of 12 men and 8 women, at the age range of 44–74 years.

**Table 2:** Clinical characteristics of the CLL patients (n = 90)

**Characteristic**	**Frequency (%)**
**Sex**	
Male	70
Female	30
**Age**, **y**	
40–49	7
50–59	7
60–69	38
70–79	39
80–89	10
**Clinical phase**	
Progressive *	47
Non-progressive *	53
**Rai Stage**	
O	39
I	26
II	7
III	26
IV	3
**Treatment**	
Previously treated **	33
Previously untreated ***	67

* For definition, see ref [43].

** Test performed at relapse / progression prior to initiation of salvage therapy.

*** Among 60 untreated patients, 33 were indolent and 27 were progressive/symptomatic but tested prior to first-line therapy.

Heparinized blood containing tumor cells was collected from patients with CLL (n = 90), MCL (n = 8), hairy cell leukemia (HCL) (n = 2), B-cell prolymphocytic leukemia (B-PLL) (n = 2), T-cell prolymphocytic leukemia (T-PLL) (n = 1), chronic myelogenous leukemia (CML) (n = 7), acute myelogenous leukemia (AML) (n = 7), pre B-cell acute lymphoblastic leukemia (pre B-ALL) (n = 1), follicular lymphoma (FL) (n = 2) and lymphoplasmacytoid lymphoma (n = 2). Bone marrow tumor cells were obtained from patients with multiple myeloma (MM) (n = 6). Blood was also drawn from healthy control donors (n = 20).

This study was approved by the regional ethics committee (http://www.epn.se) and all samples were collected with informed consent of the patients.

### Isolation of cells

Peripheral blood mononuclear cells (PBMC) from normal donors and leukemic cells from blood and bone marrow were isolated using Ficoll-Paque Plus (GE Healthcare, Buckinghamshire, UK) density-gradient centrifugation. T and B lymphocytes were purified by negative selection using MACS beads (Miltenyi Biotec, Bergisch Gladbach, Germany) according to the manufacturer’s instructions. The purity of the isolated populations was tested by direct immunofluorescence using monoclonal antibodies against CD3, CD19, and CD14 (BD Biosciences, San Jose, CA, USA). The purity was > 90% for normal B cells and > 70% for normal T cells.

### CLL cell lines

Six cell lines of CLL origin were also studied. The cell line EHEB was obtained from the German collection of Microorganisms and Cell Cultures (DSMZ, Braunschweig, Germany) while the others (I83-E95, 232-B4, WaC3-CD5, CII and JCA) [45,46] were a kind gift from professor Anders Rosén (Linköping University, Sweden) and professor Kenneth Nilsson (Uppsala University, Sweden). The cell lines were cultured in RPMI-1640 medium supplemented with 10% fetal bovine serum (FBS), L-glutamine (2 mM), penicillin (100 U/mL), and streptomycin (100 μg/mL) (Gibco, Paisley, Scotland).

### RT-PCR and quantitative PCR (q-PCR) amplification of OPTC mRNA

Total RNA was extracted from tumor cells and normal PBMCs using PureLink^TM^ RNA mini kit (Ambion, Carlsbad, CA, USA) according to the manufacturer’s instructions. First strand cDNA was synthesized using 2 μg total RNA in 20 μL reaction mixture consisting of 4 μL 5× reaction buffer, 1 μL dNTP (10 mM), 1,5 μL 100 uM dithiothreitol (DTT) 1 μL 10 pmol/mL random hexamer (N6), and 200 U M-MLV reverse transcriptase (Gibco). The mixture was incubated at 42°C for 50 minutes. PCR amplification was performed using OPTC-specific primers TTGACCTCTCCAACAACCTC (sense) and GGTCACAGAAGACGTCTCTC (antisense) [9]. A 25 μL reaction mixture was prepared using 2,5 μL 10× buffer, 3 μL 25 mM MgCl_2_, 1,5 μL dNTP (10 mM), 5 pmol of each primer, and 1 U Ampli-Taq Gold DNA polymerase (Perklin-Elmer/Applied Biosystems, Boston, MA, USA). PCR was initiated by 10 min at 95°C, followed by 38 cycles at 94°C for 60 seconds and 60°C for 90 seconds leading to a 327 bp amplicon. PCR products were visualized by running agarose gel electrophoresis containing ethidium bromide.

Quantitative q-PCR was performed using three Taqman gene expression assays for OPTC (Applied Biosystems, assay ID# Hs01057359_m1, Hs01057360_m1 and Hs01057361_m1). PCR reactions consisting of 1 μL cDNA, 1× TaqMan® Universal PCR Master Mix, 0.2 μM TaqMan® primer-probe mix were carried out in final volumes of 20 μL using an ABI Prism 7900 Sequence Detection System (Applied Biosystems). Reactions were initiated with 10 min incubation at 95°C followed by 40 cycles of 95°C for 15 sec and 60°C for 60 sec. The analysis was based on individual samples run in triplicates for each of the three OPTC assays, of which one mean Ct value was calculated. The expression of OPTC was then normalized against the housekeeping gene GAPDH (Applied Biosystem assay #Hs02758991_g1) and relative quantities were determined by the comparative Ct (ΔCt) method.

### Production of OPTC protein in bacteria, yeast, and mammalian cells

Due to lack of commercially available OPTC protein, we established an expression system based on a human full-length precursor OPTC transcript that was amplified by PCR using a pool of CLL cDNA from 10 patients as template. PCR amplification was performed using OPTC specific primers GAATTCATGAGGCTCCTGGCTTTCCTGAGTC (sense) and GCGGCCGCTCTACGTGAAGCGGCCGATGG (antisense). PCR reaction mixture was prepared using 2.5 μl of 10× buffer, 2 μl of 25 mM MgCl_2_, 1.5 μl dNTPs (10 mM), 5 pmol of each primer and 1 unit of Ampli-Taq Gold DNA polymerase (Perkin-Elmer/Applied Biosystems). PCR was initiated by 1 cycle at 95°C for 10 min, followed by 35 cycles at 92°C; 30 sec, 60°C; 30 sec, and 72°C; 90 sec. The PCR products were cloned into pGEM-T easy vector (Promega, Madison, WI, USA) and subcloned into *Eco*RI-*Not*I site of pcDNA3.1+ vector (Invitrogen, Carlsbad, CA, USA) for mammalian expression. For bacterial expression, this vector was slightly modified by insertion of ribosome binding sequences (RBS). The *Eco*RI-*Not*I sequence was also subcloned into pGAPZα-A vector for yeast (*P*. *pastoris*) expression (Invitrogen). The recombinant plasmids were selected for sequencing. For bacterial expression, an in-frame clone was selected and transformed into *E*.*coli* strain JM109. The bacteria culture was harvested after 24 h and a bacteria pellet was collected by centrifugation. A small volume of phosphate buffered saline (PBS) was added to the pellet and cell lysate was prepared by 5× 15 sec of sonication. The yeast supernatant was collected after 72 h of culture and concentrated up to 30 times using Amicon Ultra-15 Centrifugal Filter Units (Millipore Corporation, Bedford, MA, USA). For expression of OPTC in mammalian cells, the EcoRI-NotI pcDNA3.1+ vector was transiently transfected into mouse TM3 cell line. After 48 h, cell lysate was prepared as described below. Yeast supernatant, bacteria and TM3 cell lysate were subjected to Western blot.

Recombinant bovine OPTC, produced in 293-EBNA cells, was prepared as described previously [8,13]. Bovine OPTC is 75% homologous to human OPTC (HomoloGene, http://www.ncbi.nlm.nih.gov/) and served as a positive control in Western blot and deglycosylations experiments (see below).

### Anti-OPTC polyclonal antibodies

In the absence of commercially available anti-OPTC antibodies recognizing OPTC in CLL, a rabbit anti-OPTC polyclonal antibody (pAb) was generated against a 12-mer synthetic peptide (CDPEEHKHTRRQ) purchased from Thermo Electron Corporation GmbH (Ulm, Germany) corresponding to the carboxy-terminal (C-terminal) part of human OPTC [11]. The antibody was purified by affinity chromatography. A previously characterized rabbit anti-OPTC pAb generated against the human N-terminal peptide VLNPDNYGEVIDLSNYEELTDYGDQLPEVK of OPTC was also used [8].

### Cell lysate

Cell lysates were prepared as described [17] with minor modifications from PBMC of CLL patients (n = 30) and healthy controls (n = 10) as well as the transfected cell line TM3. 50×10^6^ cells were lysed in 1 mL of buffer containing 0.2% Triton-X, 130 mM HEPES, 4 mM MgCl_2_, 10 mM EGTA with 2% proteinase inhibitor cocktail (Sigma, St Louis, MO, USA). After 1 h incubation on ice, lysates were centrifuged at 2500 rpm for 5 min and the soluble fraction (“upper fraction” holding cytosollic proteins) was collected. The Triton-X resistant pellet was dissolved in 1× NuPAGE LDS Sample Buffer (Invitrogen) and sonicated for 3x15 sec (“lower fraction” containing proteins from membrane and inner organelles). The protein concentration of the two fractions was measured by Bio-Rad Protein Assay according to the manufacturer’s instructions (Bio-Rad Laboratories, Hercules, CA, USA) and the samples were subjected to Western blot.

### Subcellular fractionation

To further analyze the cellular localization of OPTC in CLL cells, proteins were extracted according to the 4-step procedure of Qproteome Cell Compartment Kit (Qiagen, Hilden, Germany) isolating cytosolic, membrane, nuclear and cytoskeletal proteins, respectively.

Briefly, 50x10^6^ PBMC of CLL patients (n = 8) were lysed in extraction buffer 1 for 10 min at 4°C. After centrifugation at 1000 g for 10 min, the supernatant was collected as the “cytosolic fraction”. The remaining pellet was resuspended in extraction buffer 2 for 30 min at 4°C. After centrifugation at 6000 g for 10 min, supernatant was collected as the “membrane fraction”. Benzonase buffer was added to the remaining pellet and incubated for 15 min incubation at room temperature, followed by addition of extraction buffer 3 and incubation for 10 min at 4°C. After centrifugation at 6800 g for 10 min, the supernatant was collected as the “nuclear fraction”. The remaining pellet was dissolved in extraction buffer 4 and collected as the “cytoskeletal fraction”. The protein concentration of each fraction was measured by Bio-Rad Protein Assay (Bio-Rad Laboratories) and the samples were analyzed by Western blotting.

### Nuclei isolation

Nuclei EZ Prep Nuclei Isolation kit (Sigma) was used to analyze nuclear localization of OPTC. Briefly, 50×10^6^ PBMC of CLL patients (n = 8) were lysed in Nuclei EZ lysis buffer and incubated on ice for 5 minutes. After centrifugation, the supernatant containing non-nuclear proteins was collected and concentrated 8× using Amicon Ultra-15 Centrifugal Filter Units (Millipore Corp.). The resultant nuclear pellet was washed in Nuclei EZ lysisbuffer, centrifuged, resuspended in Nuclei EZ storage buffer, and sonicated for 3×10 sec. The protein concentration of both fractions was measured by Bio-Rad Protein Assay (Bio-Rad Laboratories) and analyzed by Western blotting.

### Endoplasmic reticulum isolation

Endoplasmic Reticulum (ER) Isolation Kit (Sigma) was used to analyze the localization of OPTC in ER. Briefly, 800x10^6^ PBMC of CLL patients (n = 3) were suspended in 1× hypotonic isolation buffer and lysed with a Dounce homogenizer (Sigma). After centrifugation at 1000 g for 10 min, the resultant pellet enriched in cell nuclei [47] was suspended in 1× sample buffer (Invitrogen) and sonicated for 3×10 sec. The supernatant was further clarified by centrifugation for 15 min at 12 000 g and then at 100 000 g for 60 min. The final supernatant (holding cytosolic proteins) was collected and utilized for analysis. The remaining pellet containing the crude microsomal fraction, which included the rough and smooth ER, was suspended in isotonic extraction buffer and homogenized using a pellet pestle (Sigma). The protein concentration of the three fractions was measured by Bio-Rad Protein Assay (Bio-Rad Laboratories) and analyzed by Western blotting.

### Chemical deglycosylation of the OPTC protein

Trifluoromethanesulfonic acid (TFMS) removes all carbohydrates chains from glycoproteins regardless of linkage and composition [48]. Two sources of recombinant OPTC protein were used for chemical deglycosylation with TFMS (Sigma) and anisole (Fluka, Sigma): 1) recombinant human OPTC expressed in yeast and 2) recombinant bovine OPTC expressed in 293-EBNA cells. 250 μl of 1× OPTC yeast culture and 30 μg of bovine OPTC were precipitated separately by incubation in 99,7% ethanol at −20°C over night. After centrifugation at 15 000 g for 20 min, the protein pellets were washed in 95% ethanol, collected by centrifugation and dried in a vacuum centrifuge for 20 min. Three pellets were prepared for each sample and were added 1) 200 μl TFMS for 30 min, 2) TFMS/anisole (9:1) for 1 h and 3) TFMS/anisole (9:1) for 3 h. The samples were kept on ethanol/dry ice during the addition of TFMS/anisole and on ice for the remaining incubation time. The reaction was stopped by adding 2 M Tris base (pH 8) until pH reached 6. The samples were dialyzed against 10 mM phosphate buffer for 24 h, concentrated up to 20 times using Amicon Ultra-15 Centrifugal Filter Units (Millipore Corp.), and then subjected to Western blot.

### Western blot

Cell lysate (20 μg), serum (1:50), yeast supernatants, bacterial lysate (10 μg) and recombinant bovine OPTC (0,5 μg) were separated on a 10% NuPAGE Bis-Tris gel (Invitrogen) at 120 V for 3 h under reducing conditions. Resolved proteins were transferred onto Immobilon-P PVDF membrane (Millipore Corp.) in a Mini-Transblot Cell (Invitrogen). Non-specific antibody binding was blocked by incubating the membranes at room temperature for 1,5 h with 5% non-fat milk (Semper, Stockholm, Sweden) in PBS plus 0.05% Tween 20 (PBS-T). The membranes were incubated with 10 μg/ml of rabbit anti-OPTC pAbs (C- and N-terminal) over night at +4°C and a secondary horseradish peroxidase (HRP)-conjugated goat anti-rabbit antibody (DakoCytomation, Glostrup, Denmark) for 1.5 h at room temperature. Both incubations were followed by 4 × 15 min washings in PBS-T. Antibody-reactive bands were visualized using Amersham Enhanced Chemiluminescence ECL™ system (GE healthcare). All Western blot figures are showing the results of our anti-OPTC C-terminal pAb but all results were confirmed by the anti-OPTC pAb provided by professor Bishop.

As a control for the amount of protein loaded, membranes were stripped of bound antibody using a buffer containing 62.5 mM Tris–HCl, 2% SDS, 100 mM Mercaptoethanol (Sigma) at 50°C for 30 min with gentle agitation. After 3×15 min washing in PBS-T, the membranes were re-probed with 2.5 μg/ml of a mouse anti-β-actin monoclonal antibody (mAb) (Sigma).

### Statistical analysis

The results of OPTC mRNA expression using qPCR are shown as mean ± SEM and statistical analysis was performed with a T-test or Anova multivariant analysis together with post hoc Tukey test.

## Competing interests

The authors declare that they have no competing interests.

## Authors’ contributions

Contribution: EM designed and performed most of the experiments, analyzed the data and wrote the paper. HR designed the project. HM provided clinical material, designed the study and wrote the paper. AÖ provided clinical material, analyzed the results and wrote the paper. FS contributed in designing the study. PB and ZT-F produced antibodies and recombinant opticin. MJ-T, AM, JM and MA produced antibodies. All authors read and approved the final manuscript.
